# Role of N6-methyladenosine methylation in head and neck cancer and its regulation of innate immune pathways

**DOI:** 10.3389/fimmu.2024.1458884

**Published:** 2024-09-30

**Authors:** Luhong Cao, Guixiang Huang, Jiangang Fan, Xingren Liu, Zhiyue Ma

**Affiliations:** ^1^ Department of Otolaryngology, Head and Neck Surgery, Sichuan Provincial People’s Hospital, School of Medicine, University of Electronic Science and Technology of China, Chengdu, China; ^2^ Department of Emergency Surgery, Sichuan Provincial People’s Hospital, School of Medicine, University of Electronic Science and Technology of China, Chengdu, China; ^3^ Department of Pulmonary and Critical Care Medicine, Sichuan Provincial People’s Hospital, University of Electronic Science and Technology of China, Chengdu, China

**Keywords:** m6A methylation, TLR, cGAS-STING, NLR, innate immune, head and neck cancer

## Abstract

N6-methyladenosine (m6A) is considered the most prevalent methylation modification in messenger RNA (mRNA) that critically impacts head and neck cancer (HNC) pathogenesis and development. Alterations of m6A methylation related proteins are closely related to the progression, therapeutic effect, and prognosis of HNC. The human innate immune system activates immune pathways through pattern recognition receptors, which can not only resist pathogen infection, but also play a vital role in tumor immunity. Emerging evidence has confirmed that m6A methylation affects the activation of innate immune pathways such as TLR, cGAS-STING, and NLR by regulating RNA metabolism, revealing its potential mechanisms in the innate immune response of tumor cells. However, the relevant research is still in its infancy. This review elaborates the biological significance of RNA m6A methylation in HNC and discusses its potential regulatory relationship with TLR, cGAS-STING, and NLR pathways, providing a new perspective for in-depth understanding of the role of RNA methylation in the innate immune mechanism and therapeutic application of HNC.

## 1 Introduction

Head and neck cancer (HNC) typically refers to a group of neoplasms originating from the oral cavity, pharynx, larynx, nasal cavity, paranasal sinus, and salivary gland, including oral cavity cancer, nasopharyngeal carcinoma, laryngeal cancer, hypopharyngeal cancer, paranasal sinus cancer, salivary gland cancer, and thyroid cancer. HNC ranks the seventh most incident cancer, with an estimated 890,000 new cases in 2018 and 450,000 of these predicted to be fatal (1). In the United States, HNC accounts for 3% of all cancer cases (51,540 new cases) and results in 1.5% of all cancer deaths (10,030 deaths) (2). Head and neck squamous cell carcinoma (HNSCC) is the most common pathological type of head and neck neoplasms (3). Approximately 1.08 million new HNSCC cases are expected per year by 2030 (4). In general, the incidence of HNC shows a rising trend year by year. Early detection and comprehensive treatment can significantly improve the survival and quality of life of patients. Therefore, it is particularly important to strengthen the understanding of the relevant molecular mechanisms regulating the occurrence and development of HNC.

Innate immunity serves as the first line of defense against various external threats, consisting of a variety of complex mechanisms to rapidly and widely respond to pathogen invasion (5). Innate immunity also plays a vital role in tumorigenesis and progression by affecting the formation, growth, and metastasis of tumors, as well as the effect of immunotherapy (6). On the one hand, the innate immune system can repress the development of tumors by recognizing and clearing abnormal cells. Innate immune cells such as natural killer (NK) cells and macrophages can recognize and kill tumor cells that have undergone abnormalities, thereby preventing their constant growth and spread (7). On the other hand, the innate immune system is critical for the treatment of tumors. For example, enhancing the ability of innate immunity to attack tumors by increasing the activity of NK cells or inhibiting immunosuppressive pathways has become one of the important therapeutic strategies for tumors (8, 9).

RNA modifications usually affect RNA splicing, stability, localization, translation, and RNA-RNA/RNA-protein interactions. N6-methyladenosine (m6A) methylation is one of the most prevalent post-transcriptional modifications, which is closely related to the occurrence and prognosis of human malignancies (10–12). m6A is a modified base that exists in all RNA types, including ribosomal RNA (rRNA), transfer RNA (tRNA), and small nuclear RNA (snRNA), as well as mRNA of cellular and viral origin. Recent studies have demonstrated the important role of m6A RNA methylation in tumor innate immune response. For example, m6A methylation guarantees the homeostasis of NK cells and tumor immune surveillance. Loss of m6A methyltransferase METTL3 alters NK cell homeostasis and inhibits NK cell infiltration and function in the tumor microenvironment (13). Meanwhile, YTHDF2 deficiency impairs the antitumor and antiviral activities of NK cells (14). In addition to the regulation on NK cells, m6A methylation mediated by METTL3 can promote the activation of dendritic cells (DCs) (15). METTL3 also drives the polarization of M1 macrophages by directly methylating STAT1 mRNA (16).

Therefore, understanding the mechanism of m6A RNA methylation in innate immune response during HNC is of great significance for developing new immunotherapeutic strategies and improving the therapeutic effect of patients. Here, we comprehensively review and explore the involvement of m6A methylation in the pathogenesis and development of HNC, as well as its different mechanisms in the signal transduction mediated by different nucleic acid sensor receptors (TLR, cGAS-STING, NOD) in the innate immune response of HNC, thereby providing a new theoretical framework for the treatment of HNC.

## 2 Molecular mechanisms of m6A methylation

m6A is a dynamic and reversible methylation modification at the N6-position of adenosine. As the most prevalent internal modification on eukaryotic mRNA, m6A mainly appears in the RRACH sequences (R = A/G, H = A/C/U) near the stop codon and 3’-untranslated region (3’-UTR). m6A has been demonstrated to critically regulate various processes of RNA metabolism, such as stability, splicing, and translation (17). Typically, m6A modification is installed by m6A methyltransferases, or writers, such as METTL3/14, removed by demethylases, or erasers, including FTO and ALKBH5, and recognized by m6A-binding proteins such as YTHDF1, termed readers (18).

**Figure 1:**
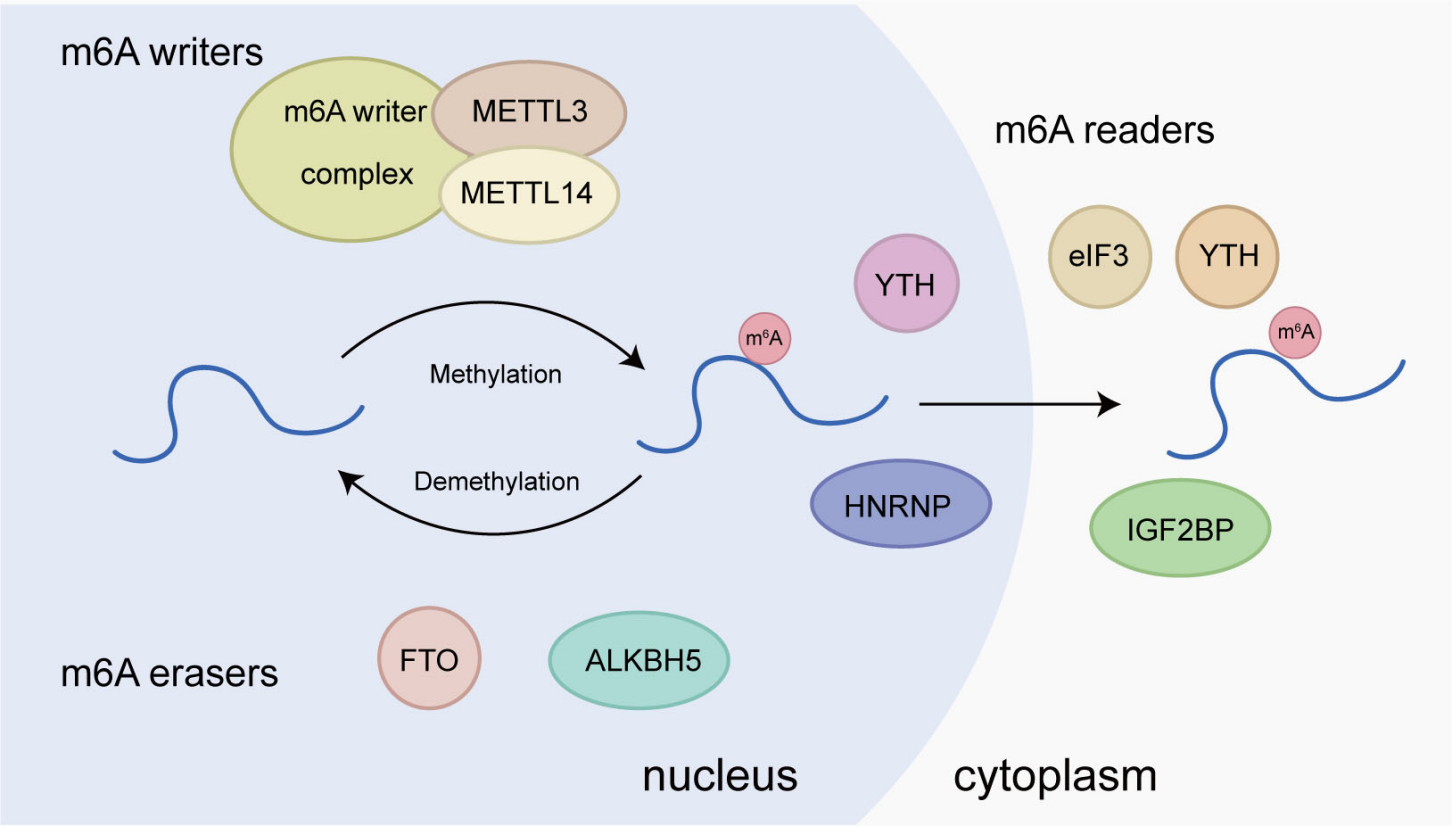
Molecular basis of m6A RNA methylation. m6A “writers”, including METTL3, METTL14, WTAP, RBM15/15B, ZC3H13, and KIAA1429, are the methyltransferases responsible for installing methyl groups on target RNAs. m6A “erasers”, including FTO and ALKBH5, are m6A demethylases. m6A “readers”, such as YTHDF1/2/3, YTHDC1, HNRNPA2B1, HNRNPC, eIF3, FMR1, and LRPPRC, are proteins that recognize m6A modification and exhibit various functions.

1. m6A writers are methyltransferases responsible for installing methyl groups on target RNAs, including METTL3, METTL14, WTAP, RBM15/15B, ZC3H13, and KIAA1429 (VIRMA) (**Figure 1**). The core component of m6A writers is the methyltransferase complex (MTC) mainly composed of METTL3, METTL4, and WTAP. METTL3 is the core subunit with catalytic activity; METTL14 has substrate recognition function, and WTAP is responsible for recruiting METTL3 and METTL14. Only when METTL3 and METTL14 form a heterodimer as the core of the MTC, the activity of METTL3 is enhanced significantly, thus activating its spatial structure) (19, 20). m6A methylation is sequence specific. m6A methylation mediated by METTL3/14 complex is preferentially enriched in RRACH (R = A or G; H = A, C, or U) motifs, which are widely used to recognize m6A methylation (21).
2. m6A erasers realize a dynamic and reversible process by removing internal m6A from RNA, in which demethylases FTO and ALKBH5 play important roles (**Figure 1**). FTO is the first demethylase discovered. Jia et al. have revealed the potent m6A demethylase activity of FTO (22). Mechanistically, FTO catalyzes the demethylation of m6A by utilizing cofactors 2-oxoglutarate and Fe2+, gradually generating N6-hydroxymethyladenosine and N6-formyladenosine (23). Rubio et al. have found that FTO preferentially demethylates m6A_m_ rather than m6A, reducing the stability of m6A_m_ mRNAs (24). Wei et al. have shown that FTO mediates internal m6A and cap m6A_m_ demethylation of polyadenylated RNA with differential substrate preferences in nucleus versus cytoplasm, in which the internal m6A demethylation correlates with changes in transcript level (25). Therefore, the subcellular localization of FTO affects its ability to perform different RNA modifications. Another demethylase, ALKBH5, is found to be highly expressed in testis. The demonstration of its demethylase activity provides the first evidence of reversible post-transcriptional modification in mRNA. ALKBH5 functions as a unique m6A regulator that specifically regulates m6A modifications without affecting other types of RNA modifications. It demethylates specific transcripts at the 3’ UTR and accelerates mRNA export from the nucleus to the cytoplasm (26).
3. m6A readers are RNA binding proteins that affect RNA splicing, nuclear transport, stability, translation, and decay by specifically recognizing and anchoring RNA with m6A modification (27). Reader proteins include YTH family, IGF2BP, HNRNPC, HNRNPA2B1, and eIF3 (**Figure 1**). YTH family proteins, including YTHDC1, YTHDC2, YTHDF1, YTHDF2, and YTHDF3, have RNA binding domains and are the first group of m6A binding proteins discovered. YTHDC1 is associated with mRNA splicing, epigenetic silencing mediated by non-coding RNA XIST, and nuclear export of mRNA (28, 29). YTHDC2, the largest member of the YTH family, also preferentially binds m6A within the consensus motif, which can improve translation efficiency while reducing the aggregation rate of its target mRNA (30). YTHDF protein exerts different functions at each end. Its C-terminal YTH domain helps it bind m6A-modified mRNA, while the N-terminal region can freely bind various cofactors to achieve expression diversity) (31). YTHDF1 was initially confirmed to bind to methylated mRNA transcripts near stop codons, and its overall distribution pattern was similar to that of m6A sites on mRNA. Mechanistic studies have revealed that YTHDF1 interacts with the translation initiation mechanism to enhance the translation efficiency of its target RNA (32). Of note, it has been unraveled that the three members of the DF protein family function together to mediate primarily the degradation of m6A-modified mRNAs, rather than to play a role in facilitating translation (33).

In conclusion, changes in the expression of m6A methylation regulators directly affect the RNA modification status of tumor cells, further modulating key biological processes such as gene expression, cell proliferation, and immune escape. In-depth studies of the way that m6A methylation regulates these biological processes can better unravel molecular mechanisms underlying the development and progression of tumors. In the clinical setting, understanding the role of m6A methylation is helpful in developing new biomarkers for tumor diagnosis, prognostic assessment, and treatment response prediction. In addition, drugs targeting m6A methylation regulators, such as METTL3 inhibitors and FTO inhibitors, are expected to become new anticancer drugs and increase the efficacy of existing treatments.

## 3 Expression and influence of m6A in HNC

**Figure 2:**
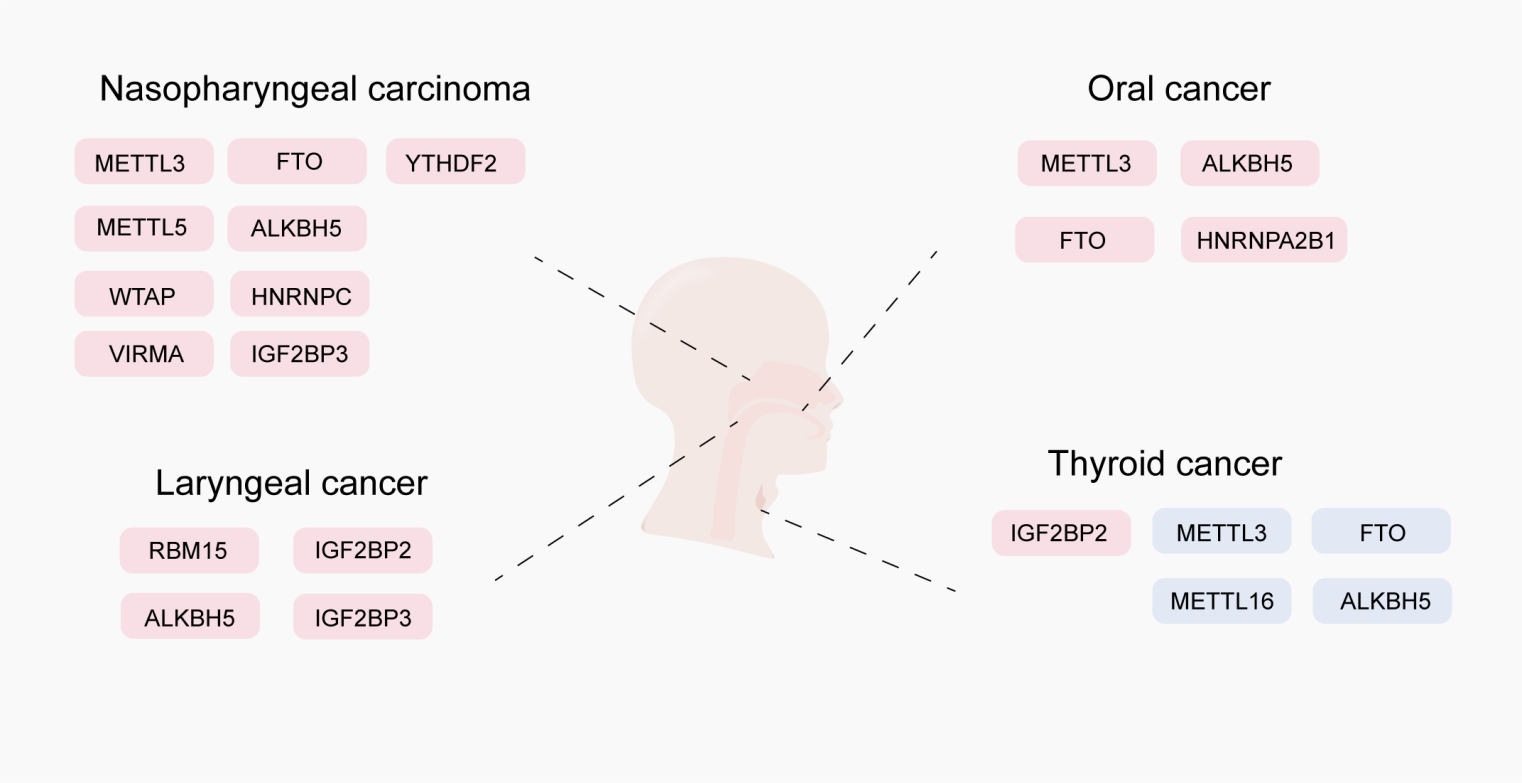
Expression and role of m6A RNA modification in head and neck cancer. This figure shows the oncogenic and anti-oncogenic effects of N6-methyladenosine (m6A) modifiers, including m6A writers (METTL3, METTL3, METTL16, VIRMA, and WTAP), m6A erasers (FTO and ALKBH5), and m6A readers (HNRNPC, IGF2BP3, IGF2BP3, YTHDF1, YTHDF2, and HNRNPA2B1). Positively regulated (upregulated) targets of m6A modifiers are labeled in red, whereas negatively regulated (downregulated) targets are labeled in blue.

RNA m6A methylation and its mechanisms are altered in a variety of HNCs, such as nasopharyngeal carcinoma (NPC), oral squamous cell carcinoma (OSCC), laryngeal squamous cell carcinoma (LSCC), and thyroid cancer (TC), leading to tumorigenesis, progression, and metastasis through dysregulation of coding and non-coding transcripts and their associated functional pathways (**Figure 2**, **Table 1**).

**Table 1:** Role of RNA m6A modification and its target genes in head and neck tumors.

Cancer type	m6A component	m6A type	State	Related gene	Related cellular activity	Reference
NPC	METTL3	Writer	increase	miR-1908-5p	Promotion of growth	(35)
NPC	METTL3	Writer	increase	SUCLG2-AS1 and SOX2	Promotion of metastasis and radiosensitivity	(34)
NPC	METTL3	Writer	increase	ZFAS1	Promotion of proliferation, migration and growth	(36)
NPC	METTL3	Writer	increase	ZNF750 and FGF14	Promotion of growth	(37)
NPC	METTL5	Writer	increase	P53^R280t^	Promotion of tumorigenesis and chemoresistance.	(38)
NPC	WTAP	Writer	increase	DIAPH1-AS1	Promotion of growth and metastasis	(40)
NPC	VIRMA	Writer	increase	E2F7	Promotion of tumorigenesis and metastasis	(39)
NPC	FTO and ALKBH5	Eraser	increase	ARHGAP35	Promotion of carcinogenic effect	(42)
NPC	FTO	Eraser	increase	OTUB1	Promotion of radioresistance	(43)
NPC	HNRNPC	Reader	increase	circITCH and miR-224-3p	Promotion of progression	(41)
NPC	IGF2BP3	Reader	increase	Notch3 signaling	Promotion of metastasis	(46)
NPC	IGF2BP3	Reader	increase	KPNA2	Promotion of proliferation and metastasis	(47)
NPC	YTHDF2	Reader	increase	TEAD4	Promotion of migration, invasion, and cisplatin resistance	(44)
NPC	YTHDF2	Reader	increase	IGF1R/AKT/S6 signaling	Promotion of radiotherapy Resistance	(45)
OSCC	METTL3	Writer	increase	SALL4 and Wnt/β-catenin signaling	Promotion of radioresistance	(48)
OSCC	METTL3	Writer	increase	FGFR3	Inhibition of anlotinib sensitivity	(52)
OSCC	METTL3	Writer	increase	MYC and HIF-1α	Promotion of proliferation, migration, oncogenicity and cisplatin resistance	(53)
OSCC	METTL3	Writer	increase	SLC7A11	Promotion of proliferation, invasion, and migration	(51)
OSCC	METTL3	Writer	increase	BMI1	Promotion of proliferation, migration and invasion	(49)
OSCC	METTL3	Writer	increase	c-Myc	Promotion of proliferation, invasion, and migration	(50)
OSCC	ALKBH5	Eraser	increase	FOXM1 and NANOG	Promotion of cisplatin resistance	(55)
OSCC	FTO	Eraser	increase	MYC and PDL-1	Promotion of proliferation, migration, and resistance to T-cell killing	(57)
OSCC	FTO	Eraser	increase	ACSL3 and GPX4	Sensitive to ferroptosis	(59)
OSCC	FTO	Eraser	increase	Cyclin D1	Promotion of progression	(56)
OSCC	FTO	Eraser	increase	eIF4G1	Inhibition of autophagy	(58)
OSCC	HNRNPA2B1	Reader	increase	FOXQ1	Promotion of proliferation	(60)
LSCC	RBM15	Writer	increase	TMBIM6	Promotion of proliferation, invasion, migration, and apoptosis	(61)
LSCC	ALKBH5	Eraser	increase	KCNQ1OT1‐HOXA9 signalling	Promotion of proliferation, invasion and metastasis	(62)
LSCC	IGF2BP2	Reader	increase	TRIM59	Promotion of proliferation and metastasis	(65)
LSCC	IGF2BP2	Reader	increase	CDK6	Promotion of invasion	(64)
LSCC	IGF2BP3	Reader	increase	TMA7	Inhibition of autophagy ; Promotion of proliferation, migration, and invasion	(63)
TC	METTL3	Writer	decrease	SETAMR	Promotion of differentiation.	(68)
TC	METTL3	Writer	decrease	LINC00894	Promotion of proliferation and lymph node metastasis	(69)
TC	METTL3	Writer	decrease	CD70	Promotion of anti-PD-1 therapy resistance	(73)
TC	METTL3	Writer	decrease	STEAP2	Inhibition of proliferation, migration, and invasion	(71)
TC	METTL3	Writer	decrease	c-rel and relA	Inhibition of growth	(72)
TC	METTL3	Writer	increase	miR-222-3p and STK4	Promotion of growth and metastasis	(70)
TC	METTL16	Writer	decrease	SCD1	Promotion of growth and lipid metabolism	(74)
TC	ALKBH5	Eraser	decrease	NRCAM	Promotion of proliferation and migration	(75)
TC	ALKBH5	Eraser	decrease	NRIP1	Promotion of glycolysis	(76)
TC	ALKBH5	Eraser	decrease	TIAM1	promoting of ferroptosis	(77)
TC	FTO	Eraser	decrease	SLC7A11	Inhibition of proliferation, migration, and invasion	(78)
TC	FTO	Eraser	decrease	APOE	Inhibition of glycolysis and growth	(79)
TC	IGF2BP2	Reader	increase	DPP4	Promotion of proliferation, invasion, migration cisplatin resistance	(80)
TC	IGF2BP2	Reader	increase	MYC	Promotion of proliferation, migration, and invasion	(82)
TC	IGF2BP2	Reader	increase	ERBB2 signaling	Promotion of tyrosine kinase inhibitor resistance	(81)

m6A binding proteins have complex regulatory mechanisms in NPC. The m6A writer METTL3 mediates m6A modification in the SUCLG2-AS1 transcript, which subsequently recognizes and stabilizes IGF2BP3. SUCLG2-AS1 regulates the expression of SOX2 by forming long-range chromatin loop, thereby regulating the metastasis and radiosensitivity of NPC (34). Moreover, METTL3 can mediate the m6A modification of miR-1908-5p (35) and lncRNA ZFAS1 (36) to affect NPC cells. METTL3 also mediates the m6A methylation of ZNF750 to decrease its expression in NPC, thereby promoting NPC progression (37). The m6A writer METTL5 enhances the translation of HSF4b to activate the transcription of HSP90B1, which binds to the gain of function p53^R280T^ protein and prevents its ubiquitination-dependent degradation, thereby promoting NPC tumorigenesis and chemoresistance (38). VIRMA is highly expressed in NPC, which elevates the expression of E2F7 in an m6A-dependent manner to promote the proliferation and metastasis of NPC cells *in vitro* and *in vivo* (39). WTAP expression is significantly upregulated in NPC, and lncRNA DIAPH1-AS1 is identified as the m6A target of WTAP. DIAPH1-AS1 acts as a molecular linker to promote the formation of MTDH-LASP1 complex and elevate LASP1 expression, ultimately promoting NPC growth and metastasis (40). m6A reader HNRNPC mediates the downregulation of circITCH to prevent miR-224-3p chelation and promote NPC tumorigenesis (41). A higher expression of FTO and ALKBH5 in tissues of patients with advanced NPC predicts a poorer prognosis. ARHGAP35 acts as a downstream target of FTO and ALKBH5 to induce NPC tumorigenesis) (42). In addition, FTO confers radioresistance to NPC cells by promoting OTUB1-mediated anti-ferroptosis (43). YTHDF2 recognizes WTAP-mediated TEAD4 m6A methylation to promote its stability and lead to abnormal upregulation of TEAD4, thereby promoting NPC migration, invasion, and cisplatin resistance (44). YTHDC2 also promotes radiotherapy resistance of NPC cells by activating the IGF1R/ATK/S6 signaling axis (45). IGF2BP3 expression is significantly increased in metastatic NPC. IGF2BP3 maintains NOTCH3 mRNA stability by inhibiting CCR4-NOT complex-mediated deadenylation (in an m6A dependent manner), thereby maintaining the activation of NOTCH3 signal and increasing the transcription of downstream genes related to stem cells, and ultimately promoting tumor metastasis (46). IGF2BP3 can also promote the proliferation and metastasis of NPC cells by affecting the stability of m6A-modified KPNA2 (47).

m6A methylation modification plays a vital role in the occurrence and development of OSCC. The m6A writer protein METTL3 exerts multiple functions in OSCC. METTL3/SALL4 axis promotes cancer stem cell (CSC) phenotype and radiotherapy resistance in OSCC via the Wnt/β-catenin signaling pathway (48). METTL3 can recognize m6A residues on BMI1 3’-UTR and bind to m6A reading protein IGF2BP1 to promote BMI1 translation, thereby inducing OSCC cell proliferation and metastasis (49). METTL3 enhances c-Myc stability through YTHDF1-mediated m6A modification to promote tumorigenesis in OSCC (50). METTL3 can also strengthen the mRNA stability of SLC7A11 through m6A-mediated IGF2BP2 binding, thereby promoting OSCC progression (51). Furthermore, m6A is implicated in the drug resistance of OSCC. METTL3-mediated FGFR3 m6A modification plays a key role in the sensitivity of anlotinib in OSCC (52). METTL3-HIF-1α-MYC establishes a positive feedback loop to promote OSCC progression, which accelerates the proliferation, migration, carcinogenicity, and cisplatin resistance of OSCC cells (53). DDX3 is a human DEAD-box RNA helicase involved in RNA metabolism and translation) (54). DDX3 directly regulates m6A through the demethylase ALKBH5, thus reducing the transcription of cancer stem cell transcription factors FOXM1 and NANOG, and eventually leading to chemotherapy resistance (55). m6A demethylase FTO can regulate the cell cycle of oral cancer and promote the cancer progression by regulating the expression of cyclin D1 (56). Arecoline-induced FTO, a high-risk factor of OSCC, elevates the stability and expression of PD-L1 transcript by mediating m6A modification and MYC activity, respectively. PD-L1 upregulation enables OSCC cells to have better proliferation, migration, and resistance to T cell killing (57). FTO can also regulate autophagy and affect tumorigenesis by targeting the eIF4G1 gene encoding in OSCC (58). In addition, high-expression of FTO in OSCC cells promotes ferroptosis by regulating ACSL3 and GPX4 (59). m6A reader protein HNRNPA2B1 is highly expressed in OSCC and enhances the malignant phenotype of OSCC by stabilizing m6A-modified FOXQ1 mRNA, which ultimately aggravates the malignancy and tumorigenicity of OSCC (60).

LSCC is a malignant tumor originated from laryngeal squamous cell lesions. The overall mRNA m6A methylation level is significantly increased in LSCC patients. RBM15, as the “writer” of methyltransferase, is notably upregulated in LSCC and associated with poor prognosis. Knockdown of RBM15 reduces the proliferation, invasion, migration, and apoptosis of LSCC *in vitro* and *in vivo* (61). ALKBH5-mediated m6A modification of KCNQ1OT1 regulates LSCC cell proliferation, migration, and invasion through the upregulation of HOXA9 (62). IGF2BP3 manipulates TMA7-mediated autophagy and cisplatin resistance in LSCC via m6A RNA methylation (63). IGF2BP2 overexpression in LSCC promotes the CDK6 mRNA stability and protein expression, thus increasing the expression of CDK6 and promoting LSCC cell proliferation and invasion *in vitro* and tumor growth in xenograft tumor models (64). IGF2BP2 can also stabilize circMMP9 in an m6A-dependent manner, and then circMMP9 recruits ETS1 to control TRIM59 transcription, thereby promoting LSCC progression via the PI3K/AKT signaling pathway (65).

Increasing evidences have revealed the close correlation between aberrant m6A modification and the occurrence and development of TC. Xu et al. have found differential expression patterns of multiple m6A-related genes between TC patients and normal population in The Cancer Genome Atlas (TCGA) dataset, including METTL3, YTHDC2, WTAP, YTHDF1, ALKBH5, METTL14, YTHDC1, FTO, ZC3H13, YTHDF2, and RBM15. By verifying the gene signatures of three Gene Expression Omnibus (GEO) datasets (GSE33630, GSE35570, and GSE60542), they conclude that m6A modification affects the prognosis of TC (66). Through bioinformatics analysis, Wang et al. have revealed that the expressions of METTL3, YTHDC1, FTO, METTL14, RBM15, YTHDF3, WTAP, ALKBH5, METTL16, YTHDC, and YTHDF1 in TC tissues are significantly lower than those in normal thyroid tissues (67). Since there are multiple subtypes of TC with different clinical and pathological characteristics, the function of m6A modification proteins in different subtypes of TC needs further study and verification. METTL3 expression in TC cells, especially in anaplastic thyroid cancer (ATC) cells, is significantly lower than that in normal thyroid cells. METTL3-mediated m6A modification controls SETMAR expression, and SETMAR significantly regulates TC cell proliferation, epithelial mesenchymal transition (EMT), thyroid differentiation related gene expression, radioiodine uptake, and sensitivity to MAPK inhibitor based redifferentiation therapy (68). METTL3 accelerates papillary thyroid cancer (PTC) progression by enhancing LINC00894 mRNA stability and upregulating LINC00894 expression via m6A-YTHDC2-dependent pathway (69). In addition, METTL3 induces miR-222-3p expression, thereby inhibiting STK4 expression and promoting the malignancy and metastasis of TC (70). Some studies have also reported that METTL3 acts as a thyroid tumor suppressor. METTL3-mediated STEAP2 m6A modification plays a tumor suppressive role in PTC by blocking Hedgehog signaling pathway and EMT (71). METTL3 cooperates with YTHDF2 through the c-Rel and NF-κB pathways to change Tan invasion and repress tumor growth, thereby inhibiting the occurrence and development of PTC (72). Overexpression of METTL3 can enhance the efficacy of anti-PD-1 therapy in PTC and ATC. Low expression of METTL3 in TC cells leads to demethylation and stabilization of CD70 mRNA in a YTHDF2-dependent manner. Subsequent upregulation of CD70 protein increases the abundance of immunosuppressive Tregs and terminally exhausted T cells, thereby inducing resistance to anti-PD-1 therapy (73). METTL16 is lowly expressed in PTC tissues, resulting in elevated SCD1 expression and enhanced lipid metabolism in PTC cells, which promote PTC progression and lead to poor clinical prognoses (74). circNRCAM is highly expressed in PTC cells, which enhances PTC cell proliferation, invasion, and migration by interacting with ALKBH5 and inhibiting miR-506-3p) (75). ALKBH5 modifies circNRIP1 to regulate PKM2 expression by competing for the sponge effect of miR-541-5p and miR-3064-5p, thereby affecting the glycolytic function of PTC cells (76). ALKBH5 overexpression inhibits TC cell proliferation, increases Fe2+ and reactive oxygen species levels, decreases GPX4 and SLC7A11 proteins, and eventually retards TC progression (77). FTO is downregulated in PTC and acts as a tumor suppressor gene. FTO reduces the expression of SLC7A11 through ferroptosis to suppress the development of PTC) (78). Meanwhile, FTO inhibits the expression of APOE via IGF2BP2-mediated m6A modification and regulates IL-6/JAK2/STAT3 signaling pathway to inhibit the glycolytic metabolism of PTC, thereby repressing tumor growth (79). IGF2BP2 is upregulated in PTC and interacts with DPP4 in an m6A-dependent manner to activate the NF-KB pathway and promote lymph node metastasis. Knockdown of IGF2BP2 increases the sensitivity of PTC cells to cisplatin to a certain extent (80). Moreover, IGF2BP2-dependent activation of ERBB2 signaling is involved in acquired resistance to tyrosine kinase inhibitors (81). By competitively binding to miR-204, lncRNA MALAT1 upregulates IGF2BP2 via m6A modification and enhances MYC expression, thereby stimulating the proliferation, migration, and invasion of TC cells, accompanied by the attenuation of tumor growth and apoptosis (82).

To sum up, aberrant m6A methylation affects the occurrence and development of HNC at all levels. Notably, the expression of some m6A writers and erasers are both upregulated in HNC, and their functions may be dependent on m6A modification. Nevertheless, the specific mechanisms and targets of these proteins may differ. Research on the specific roles and interactions of these proteins can reveal the complex regulatory mechanisms of m6A modification in diseases, thereby offering important clues for understanding the molecular mechanism of HNC and also providing guidance for future research and treatment of HNC.

## 4 Potential regulatory role of m6A methylation in innate immune pathways in HNC

Modern immunology believes that the recognition of tumor by innate immunity can be divided into antibacterial immune recognition and emergency immune recognition, but attempts to identify the molecular pathways shared by all tumors have been unsuccessful, and there is no evidence that the common determinants or patterns expressed by tumor cells can be recognized by the immune system. In contrast, immune cells can sense several types of modifications associated with various tumors, but not specific to these tumors. Nucleic acid detection is a typical example of using infection detection pathways to promote the recognition of growing tumors and trigger tumor specific immune responses, thereby coordinating downstream immune responses (83, 84). In the immune recognition of HNC, nucleic acid sensing involves toll like receptors (TLRs) (85) and cytosolic nucleic acid sensors such as stimulator of interferon genes (STING) (86) and NOR like receptors (NLRs) (87). All these immune recognition and immune response processes can be regulated by m6A methylation.

### 4.1 TLR pathway

TLRs are a class of pattern recognition receptors (PRRs) that can recognize pathogen associated molecular patterns (PAMPs) and damage associated molecular patterns (DAMPs), thereby initiating immune responses (88). When ligands appear and bind to TLRs, dimers are formed and cascade signals are generated, which subsequently initiate the MyD88 pathway or TIR domain-containing adaptor inducing IFN-beta (TRIF) pathway (89). The activation of TLR signal transduction originates from the domain of cytosolic Toll/IL-1 receptor (TIR), which interacts with MyD88, an adaptor protein contained in the TIR domain, to initiate interleukin 1R related kinase (IRAK). Tumor necrosis factor receptor-associated factor (TRAF) is a molecule that assists in sending signals from IRAK to the nucleus, which further signals to the nucleus and leads to the activation of mitogen-activated protein kinases (MAPKs) and nuclear factor-kappaB (NF-κB) (90). In the TRIF pathway, TRIF interacts with TRAF6 and TRAF3. TRAF6 recruits the receptor-interacting protein 1 (RIP1), which in turn activates the transforming growth factor beta-activated kinase 1 (TAK1), leading to the activation of NF-κB and MAPKs and the induction of inflammatory cytokines (91). In contrast, TRAF3 recruits IKK related kinases TANK-binding kinase 1 (TBK1) and inducible IkappaB kinase inhibitor (IKKi) as well as nuclear factor (NF)-kappaB essential modulator (NEMO) for interferon regulatory factor 3 (IRF3) phosphorylation. Subsequently, IRF3 forms dimers and translocates from the cytoplasm into the nucleus, thereby inducing the expression of type I interferon (IFN) (92). Previous studies have shown the crucial implication of TLRs in the occurrence and proliferation of malignant tumors. On the one hand, cancer-related TLR regulation can elevate the susceptibility to infection. For example, TLR1 SNP associated with lymphoma can indicate enhanced susceptibility to Helicobacter pylori infection in lymphoma patients (93). TLR1 polymorphism provides an additional step for Helicobacter pylori infection to induce further oncogenic transformation (93). These infections can participate in the process of tumorigenesis through the further regulation of TLRs. For example, Helicobacter pylori lipopolysaccharide has the ability to induce TLR4 expression, and this regulation leads to gastric epithelial cell proliferation via the MEK1/2-ERK1/2 MAP kinase pathway (94). Therefore, TLR activation induced by various ways facilitates the carcinogenesis process by releasing pro-inflammatory cytokines and anti-apoptotic factors, recruiting immune cells, and promoting the proliferation of cells in the tumor microenvironment (TME), thereby creating a tumor friendly environment.

TLRs are critically participate in the occurrence and development of HNC. TLR2, TLR4, and TLR9 are expressed in primary tumors, neck metastatic tumors, and recurrent tumors of oral tongue squamous cell carcinoma (OTSCC) ranging from the tumor surface to the invasive front, which may be one of the important factors to promote the invasion of OTSCC (95, 96). TLR2 is expressed on keratinocytes of OTSCC and specifically regulates the growth and survival of tumor cells by promoting immune escape and inhibiting apoptosis (97). TLR3 is overexpressed in both HNC and squamous cell carcinoma, and the TLR3 rs3775291 polymorphism mutation genotype is associated with poor survival in patients with oral cancer (98). TLR4 is also a key mediator in the tumorigenesis of HNC. TLR4 expression is upregulated in oral cancer, and elevated TLR4 expression drives the transition of epithelial cells to mesenchymal cells, thereby promoting metastasis, differentiation, and proliferation, and resulting in poor survival and exacerbating disease severity (99). TLR9 promotes the migration of oral cancer cells HB *in vitro* through the activation of its cognate ligand CpG ODN (100). In contrast, OSCC patients with higher levels of TLR9 but not TLR2 or TLR4 present much worse 10-year overall survival (101). In addition, various types of TLRs are also highly expressed in NPC, LSCC, and TC (101–104).

**Figure 3:**
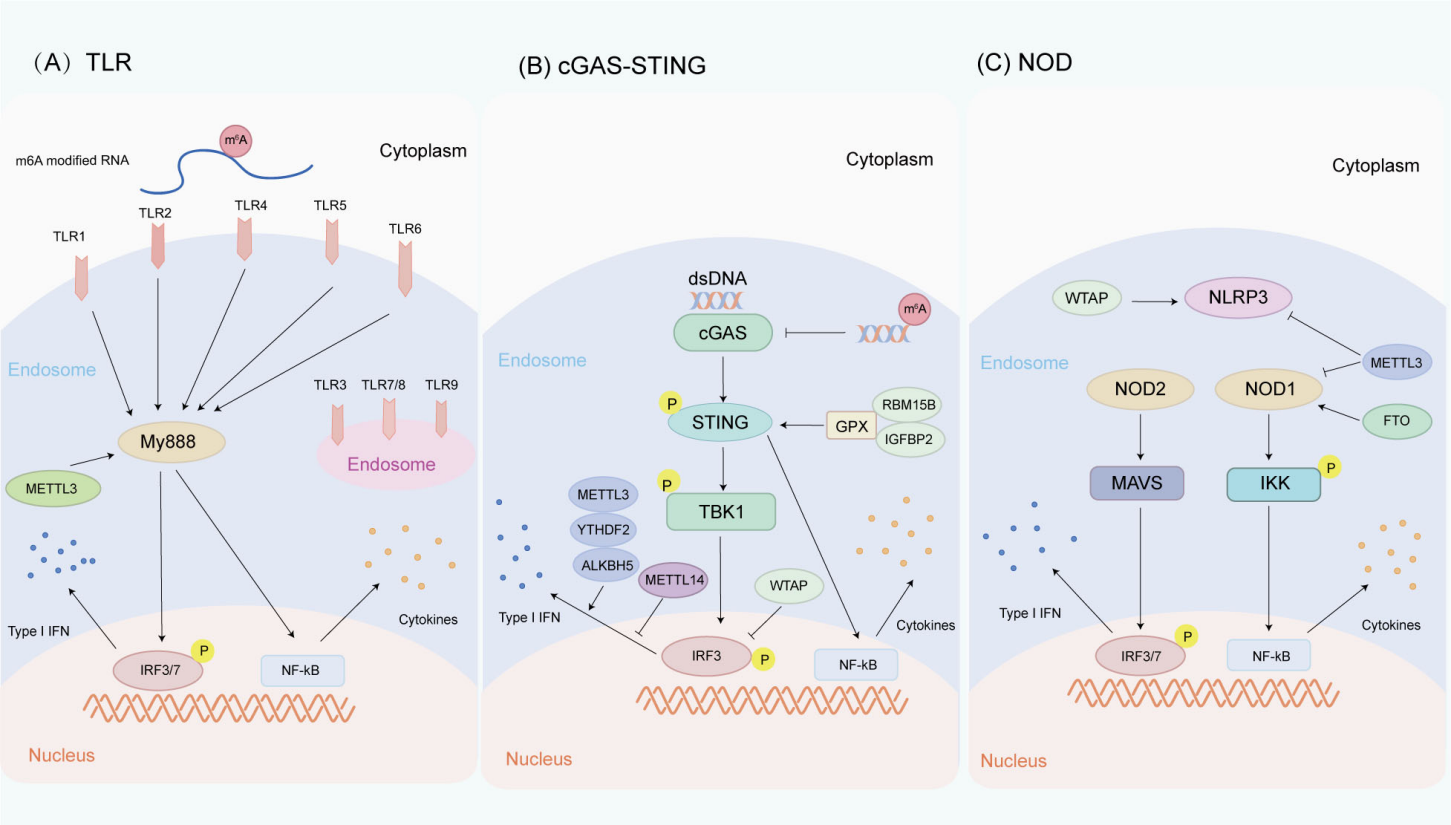
Innate immune pathways are regulated by m6A methylation in HNC. **(A)** Relationship between m6A methylation and TLR pathway. TLR1, TLR2, TLR4, TLR5, and TLR6 are located on the cell surface, while TLR3, TLR7, TLR8, and TLR9 are located in the endosomal/lysosomal part. METTL3 regulates the alternative splicing of MyD88, a participant in the TLR-mediated antiviral immune pathway. In addition, the difference between m6A-modified RNA and unmodified RNA can cause different TLR signaling activities. **(B)** Relationship between m6A methylation and cGAS-STING pathway. cGAS is an intracellular DNA sensor that activates the IFN pathway by recognizing dsDNA in the cytoplasm. RBM15B and IGFBP2 activate the cGAS-STING pathway by methylating GPX4 mRNA, thereby increasing the activity of the cGAS-STING pathway. METTL3, YTHDF2, AKLBH5, METTL14, and WTAP regulate the production of type I IFNs triggered by dsDNA. **(C)** Relationship between m6A methylation and NLR pathway. NLRs are a class of intracellular pattern recognition receptors. METTL3 and FTO regulate NOD1-mediated NF-κB signal transduction WTAP promotes NLRP3 mRNA m6A methylation, thereby upregulating NLRP3 inflammasome activation, while knockdown of METTL14 inhibits NLRP3 inflammasome activation.

Tong et al. have demonstrated that METTL3 is a positive regulator of the innate response of macrophages. Mechanistically, the transcript of Irakm gene, which encodes a negative regulator of TLR4 signaling, is highly m6A modified. METTL3 deletion abates m6A modification on Irakm mRNA and slows down its degradation, resulting in the increase of Irakm expression, which ultimately inhibits TLR signal-mediated macrophage activation and promotes tumor growth (105). Knockdown of the methyltransferase METTL3 regulates the alternative splicing of MyD88, a participant in the TLR-mediated antiviral immune pathway, resulting in the generation of the MyD88 splice variant, which is known to inhibit the production of inflammatory cytokines (106). Depletion of METTL3 reduces the accumulation of inflammatory cytokines and inhibits the activation of NF-κB and MAPK signaling pathways (**Figure 3A**). By comparing m6A-modified RNA and unmodified RNA, it is found that the m6A-modified RNA does not induce TLR3 transformed cells to produce detectable levels of IL-8, but also inhibits the signal transduction activity by TLR7 and TLR8 (107), while unmodified RNA can activate all these human TLRs. The inhibition of RNA-mediated immunostimulation is directly proportional to the number of modified nucleosides present in RNA. Moreover, the presence of m6A modification reduces RNA-mediated activation of monocyte-derived dendritic cells (DCs) more effectively than m5C or Ψ modification (106, 107). Briefly, m6A methylation may have complex and critical effects on TLRs and HNC pathogenesis, providing important insights into the immune mechanism of HNC and the development of therapeutic strategies for HNC.

### 4.2 cGAS-STING pathway

The cGAS-STING pathway is a sensor for cytosolic double stranded DNA (dsDNA) in central cells that allows innate immunity to respond to infection, inflammation, and cancer (108, 109). cGAS recognizes a variety of cytosolic dsDNA, including DNA with viral, apoptotic, exosomal, mitochondrial, micronucleus, and retroelement sources. cGAS binds directly to dsDNA and subsequently catalyzes the production of cyclic GMP-AMP (cGAMP) (110). After the cGAMP of stimulation, STING dimers translocate from the endoplasmic reticulum to perinuclear microsomes through the Golgi apparatus to recruit and activate TBK1, further phosphorylating IRF3 and upregulating the expression of type I IFN (111). In addition, STING can activate the NF-κB pathway in activated B cells by binding to IκB kinase (IKK) and NF-κB inducible kinase (NIK) (112). The activated NF-κB pathway cooperates with the TBK1-IRF3 pathway to induce the expression of type I IFNs (113). Type I IFNs have a variety of immunostimulatory functions, which can promote the maturation, migration, and activation of immune cells such as DCs, T cells, and NK cells (114). The possibility of DNA exposure to cytosolic DNA sensors increases in tumor cells, which induces the production of IFN and wakes up the host immune response mediated by the infiltration of immune cells (such as T cells and NK cells), thys inhibiting the progression of tumor cells (115). The cGAS-STING sting pathway downregulates the expression of anti-apoptotic protein BCL2 and upregulates the abundance of pro-apoptotic protein BAX (116). BAX-mediated mitochondrial outer membrane permeabilization and simultaneous caspase-9-driven caspase-3 activation contribute to apoptosis (117). Therefore, the intact cGAS-STING is an important regulator of cancer cell growth and immune surveillance. Due to selective pressure, surviving cancer cells often have defects in the cGAS-STING pathway. It has been detected that the activation of cGAS-STING is often impaired by epigenetic hypermethylation in a variety of cancers (118). All these findings suggest that understanding the regulatory mechanism of cGAS-STING pathway and its alternations in different cancer types is crucial for developing new immunotherapeutic strategies.

Human papillomavirus (HPV) is closely associated with HNSCC, and HPV16 is the primary high-risk HPV type (119). In HPV16-positive HNSCC cells, the cGAS-STING pathway is blocked due to the highly conserved LXCXE motif of its 16E7 oncogene, which in turn leads to the blockade of the relevant immune response (120, 121). In HNSCC, STING expression is also associated with enhanced immune infiltration in the tumor microenvironment and hence is considered as a favorable prognostic factor (122). By analyzing the STING expression of HNSCC cohort in TCGA, it is found that low STING mRNA is indicative of poor overall survival (123). HNSCC patients often receive DNA damage therapy (i.e., radiotherapy and cisplatin), and data show that low STING expression is associated with treatment resistance (123). In TCGA, the prognosis of OSCC patients with high expression of cGAS-STING is better (124). In multiple solid tumor types, impaired cGAS-STING signaling is associated with poor prognosis and low immune cell infiltration. However, some studies have found the specific increase of STING expression in intrinsic tumor cells with the progression of LSCC, although no correlation between STING and CGAs has been observed at the protein level (125). The low expression of MYC in HNSCC induces DNA damage related cGAS-STING pathway, which increases the recruitment of chemokines by CD8 T cells, promotes tumor immune response, and inhibits the proliferation and migration of HNSCC (126). In NPC patients, TRIM21 promotes the degradation of VDAC2 via K48-linked ubiquitination, which inhibits pore formation by VDAC2 oligomers for mitochondrial DNA (mtDNA) release, thereby inhibiting type I IFN responses following radiation exposure (127). Therefore, understanding the activation of cGAS-STING pathway in HNC is conducive to formulating suitable immunotherapy plans.

HPV infection (oropharyngeal carcinoma) and EB virus infection (nasopharyngeal carcinoma) are the high-risk factors of HNC. m6A modification has been reported to regulate cGAS-STING signal transduction. Rubio et al. observed that the m6A methyltransferase METTL14 and demethylase ALKBH5 controlled the production of type I IFNs triggered by dsDNA (128) (**Figure 3B**). Roni et al. have revealed that m6A acts as a negative regulator of IFN response by determining the rapid turnover of IFN mRNA and thus promoting viral transmission (129). Ge et al. have found that WTAP, as one of the “writers” of m6A, participates in the intracellular WTAP-IRF3/IFNAR1 axis as a negative feedback pathway to fine tune the activation of type I IFN signaling (130). Melania et al. have unveiled that the cytoplasmic recognition of m6A methylated dsDNA enhances the activation of macrophages and dendritic cells, which may be due to the fact that m6A methylation changes the immunogenicity of cytoplasmic DNA, affects the secondary structure of DNA, and causes DNA bending, thus affecting the binding affinity between dsDNA and cGAS and promoting cGAS dimerization or its enzymatic activity (131). Chen et al. have found that RBM15B and IGFBP2 mediate m6A-modified GPX4 to promote cGAS-STING signaling activation by maintaining redox homeostasis in colorectal adenocarcinoma, thereby promoting its anticancer immune response (132). The newly discovered nuclear DNA sensor hnRNPA2B1 is also an m6A reader that can partially promote DNA-induced innate immunity by promoting mRNA m6A modification of cGAS, IFI16, and STING (133). TRIM29 is also an important innate immunomodulator, which upregulates TRIM29 translation in cisplatin-resistant ovarian cancer cells through YTHDF1-mediated m6A modification (134). Prior studies showed that TRIM29 could promote DNA and RNA viral infections by modulating DNA and RNA sensor-mediated type I interferon pathways (135, 136). A recent study also revealed that TRIM29 enhanced PERK-mediated immune responses to endoplasmic reticulum stress to facilitate viral myocarditis caused by cardiotropic viruses (137). Given the critical role of TRIM29 in regulating innate immunity and cancer, it is a worthwhile direction for in-depth research to explore whether m6A modification can regulate TRIM29 expression to influence innate immune responses in HNC. These findings highlight the important impact of m6A methylation on the cGAS-STING pathway in HNC and indicate its potential as a hotspot for future HNC research.

### 4.3 NOD pathway

NLRs are a class of intracellular pattern recognition receptors (PRRs) that recognize PAMPs or DAMPs in the cytosol and activate NF-κB complexes, initiate the assembly of a variety of protein complexes including inflammasomes, and lead to the secretion of pro-inflammatory and chemotactic cytokines (138, 139). NLRs usually consist of three domains, including an N-terminal regulatory domain, a nucleotide oligomerization binding domain (NOD), and a C-terminal leucine-rich repeat domain (LRR) (140). The combination of five different functional elements in the N-terminal domain, namely the acidic transactivation domain (AD), pyrin domain (PYD), caspase recruitment domain (CARD), death effector domain (DED), and baculovirus inhibitor repeat domain (BIR), divides NLRs into five subfamily members: NLRA, NLRB, NLRC, NLRP, and NLRX (141). The NLRP receptor subfamily consists of 14 members characterized by the presence of an N-terminal pyrin (PYD) effector domain (142), which has conserved sequence motifs found in more than 20 human proteins. NLRP1, NLRP3, and NLRC4 can assemble a multi-molecular complex called “inflammasome”. The formation of inflammasomes leads to caspase-1 activation and IL-1 family cytokine maturation, thereby transmitting apoptotic and inflammatory signals. Deletion of NLR family members including NOD1, NOD2, NLRP3, NLRC4, and NLRP12 also accelerates carcinogenicity (143–145). However, the overexpression of NLR pathway components mediates the occurrence of inflammation and also drives cancer. Some studies have directly shown that NLRs can participate in immune monitoring (146, 147), and NLRs are also related to antitumor immunity through the link between IL-18 and increased NK cell antitumor activity (148, 149). Therefore, NLRs mediate the balance between inflammation and repair to maintain the homeostasis of each tissue, and malignant tumors may occur if tilted in either direction.

Biopsy results of nasal cancer and HNC have shown that cancer cells first show consistent and strong expression of NOD1 and NAIP, while normal epithelial cells show a broader NLR spectrum with the presence of NOD1, NOD2, NLRP1, NLRP3, and NAIP (150). NOD1 stimulation induces an immune response in tumor cells different from that in normal epithelial cells (150). NOD1 and NOD2 genes are expressed in human OSCC cell line YD-10B, which may trigger immune responses via the MAPK pathway. Stimulation with the NOD2 agonist MDP can inhibit cell growth by inducing apoptosis (151). NLRP3 inflammasome acts as a negative regulator of tumorigenesis in HNSCC (152, 153), and blocking NLRP3 inflammasome can also delay the tumor-bearing speed in HNSCC mice (153). Therefore, the regulation of NOD signal transduction possesses significant potential in the treatment of HNC.

m6A RNA methylation plays a pivotal role in the regulation of NOD1 signaling pathway. Cai et al. have found that the NLP signaling pathway may be a target of METTL3. During LPS-induced inflammatory response, METTL3 deletion upregulates the NOD1 pathway in macrophages, but does not affect the NOD2 pathway. METTL3 knockdown increases the mRNA stability of NOD1 and RIPK2 in a YTHDF1- and YTHDF2-dependent manner, thereby activating the NOD1 pathway (154). Xu et al. have noted that knockdown of METTL3 in HepG2 cells significantly increases the mRNA expressions of NOD1, RIPK2, NFKBIA, and NF-κB p65 mRNA, while FTO overexpression significantly increases the mRNA expressions of NOD1 and NF-κB p65 and decreases the mRNA expression of NFKBIA (155) (**Figure 3C**). Cao et al. have verified that NLRP3 is a downstream target of METTL14 by m6A RNA immunoprecipitation, and knockdown of METTL14 inhibits the activation of NLRP3 inflammasome and alleviates acute lung injury *in vivo* and *in vitro* (156). Lan has found that WTAP promotes NLRP3 mRNA m6A methylation, thereby activating NLRP3 inflammasome and further inducing pyroptosis and inflammatory responses of HK-2 cells (157). Overall, these studies clarify the role of m6A in regulating NLR-mediated signal transduction in different diseases, while its specific role in NLR-mediated transduction in HNC needs further exploration.

## 5 Challenges and prospects

Despite considerable progress in understanding the regulation of m6A methylation on innate immune pathways, the role and mechanism of m6A methylation in different tumors remain elusive due to the high heterogeneity of tumor cells. By virtue of constantly developing technologies, such as high-throughput sequencing and single-cell analysis, we are expected to have a deeper understanding of the distribution of m6A methylation in a variety of HNCs and its impact on innate immune pathways. Further research on the mechanism of m6A methylation in tumors, particularly its regulatory effect on the innate immune pathway, can reveal the mechanism of immune escape in tumors and provide theoretical support for the development of novel immunotherapies in the clinic. For example, targeting m6A methylation regulators can enhance the recognition and clearance of tumor cells by the immune system, thereby increasing the efficacy of immunotherapy. In addition, further research also provides a basis for the discovery of new biomarkers, which can contribute to better tumor diagnosis and prognostic assessment. Nowadays, a growing number of small molecules are emerging as potential drugs targeting m6A regulators for cancer treatment. For example, corresponding competitive inhibitors, such as the METTL3 inhibitor UZH1a, were designed with virtual screening based on adenosine, a substrate analog of m6A regulators (158). Components with an m6A inhibitory activity, including the FTO inhibitor emodin (159) and the METTL3 inhibitor quercetin (160), were identified from natural products. Based on their old use, some clinical drugs, metformin, have also been found to have an m6A inhibitory activity (161). These small molecule modulators of m6A regulators target the epigenetic level and precisely intervene in RNA modification, effectively disrupting the survival mechanism of tumor cells and providing a new path for cancer therapy.

In conclusion, it is crucial for the development of new therapeutic strategies to investigate the mechanism of m6A methylation in tumor innate immune pathways. At present, research on the role of m6A methylation in innate immune pathways during HNC is still in its preliminary stage. Accordingly, it is necessary to further investigate the mechanism by which m6A methylation affects tumor recognition by innate immunity, which is crucial for promoting the understanding of tumor-immune interactions and developing new therapeutic strategies.
